# Untapped: the 1891 hop yield record

**DOI:** 10.3389/fpls.2025.1539239

**Published:** 2025-04-03

**Authors:** William L. Bauerle

**Affiliations:** Department of Horticulture and Landscape Architecture, Colorado State University, Fort Collins, CO, United States

**Keywords:** beer, carbon dioxide, climate, ecozone, gibberellic acid, Humulus lupulus

## 1 Introduction

In 1891, an article in the New York Times reported a record setting “enormous” hop harvest in Kent, Washington (Anonymous, 1891a). Kent, aptly named after the renowned English hop-growing region, established itself as the “Hop Capital of the West” during the late 19th century (Meeker, 1883). From 1870-1900, Washington’s Puget Sound ecozone was the United States leading hop producer (United States Bureau of the Census, 1902). The average dry hop yield during this period was a substantial 1,793 kg/ha, significantly surpassing the yields of major competitors like New York, England, and Germany, which averaged a mere 729 kg/ha (Anonymous, 1891a, 1891b). Astoundingly, the Puyallup Hop Company documented an unprecedented dry hop yield in 1891 of 6,268 kg/ha (Anonymous, 1891a). According to a New York Times article, the state average yield for 1891 (2,242 kg/ha) was 449 kg/ha more than the historical average (Anonymous, 1891b). Given advances in hop growing technology since that time, it is worthwhile to note that Washington’s dry hop yield in 2023 averaged 2,146 kg/ha, which is comparable to the 1870 - 1900 average, but three times lower than the 1891 record. The factors behind this historical anomaly have remained elusive for over a century. However, recent reports emerging between 2021 and 2024 offer a potential explanation. By synthesizing these reports, we piece together the unique confluence of events that may have contributed to this remarkable production anomaly. Further research is necessary to definitively confirm the contributing factors. Nevertheless, the Puyallup Hop Company’s 1891 achievement serves as a testament to the potential for exceptional yields under specific circumstances.

What do we know now that will help us unravel this one-time productivity record? Recent studies point to the roles of climate, atmospheric CO_2_ concentrations, and gibberellic acid as critical determinants of hop productivity. While other factors like soil nutrient and water properties are critical, these likely remained relatively constant across consecutive years. Delving into the roles of climate, atmospheric CO_2_ concentrations, and gibberellic acid, alongside a critical analysis of circumstantial evidence, advances a plausible explanation for the factors that might have converged in 1891.

## 2 Climate

Climate is an essential component for hop production (e.g., Zattler and Jehl, 1962; Mozney et al., 2009; Krofta et al., 2017; Forester and Schüll, 2020; Potopová et al., 2021). A large hop yield necessitates a combination of climate factors free of seasonal/daily extremes: cool growing temperatures, plentiful sunlight, a moist atmosphere, and an ample water supply (e.g. Neve, 1991; Rybaček, 1991). During the summer months, the Pacific maritime ecozone of Puget Sound historically provided such a combination of climate attributes. For hops, elevated temperatures have a pronounced effect on decreasing photosynthesis and increasing respiration (Bauerle and Hazlett, 2023). The cool summer temperatures and plentiful sunlight conditions of the Puget Sound ecozone were optimal for increasing hop photosynthesis and decreasing organ respiration (Bauerle and Hazlett, 2023). Furthermore, cool moist oceanic air flowed easterly inland, blowing straight into the region’s coastal mountains. The cool air, laden with moisture, sank. As it descended, the moisture released and became precipitation, creating a plentiful water supply. As such, the Puget Sound ecozone provided a near perfect climate and deep nutrient rich soil for hop production (e.g. Meeker, 1883; Hipple, 2011).

## 3 Atmospheric CO_2_ concentrations

Elevated atmospheric CO_2_ concentrations have been shown to significantly improve hop net carbon gain. In a controlled environment study, uptake increased as much as 60% at concentrations 285 ppm above 2021 ambient levels (Bauerle, 2021; 2023). Additionally, quantum efficiency was enhanced, lowering the leaf light compensation point by 250 µmol m^-2^ s^-1^, which largely increased carbon assimilation in the most fructiferous portion of the hop canopy (Bauerle, 2021). Why is it plausible elevated CO_2_ concentrations played into the 1891 hop yield record? Seattle and Tacoma experienced remarkable growth in the 1880s and 1890s, partly attributable to the second industrial revolution. Historical documentation indicates a substantial presence of CO_2_-emitting industries in Puget Sound during this period (La Roche, 1891; Vassault, 1892). Amidst this industrial landscape were agricultural acre-tracts, including the plot that yielded a record hop harvest in 1891. This scenario bears resemblance to CO_2_ Free-Air Concentration Enrichment (FACE) experiments, utilized by contemporary plant scientists to elevate atmospheric CO_2_ levels. Relative to ambient global concentrations, FACE experiments notably augment CO_2_ levels in open-air treatments.

**Figure 1:**
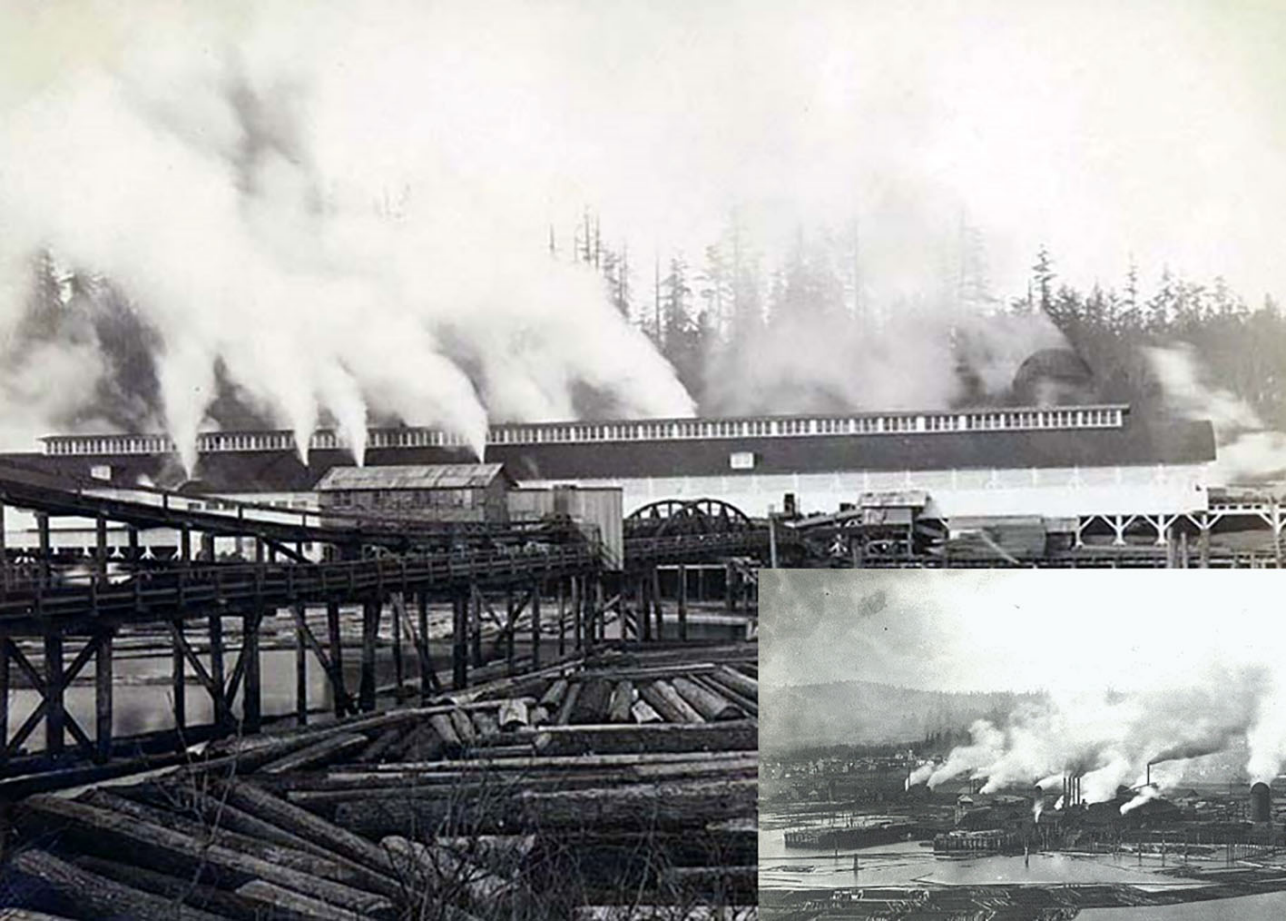
An 1891 picture taken by Frank La Roche illustrates one of the hundreds of Puget Sound CO_2_ emitting sawmills. The inset picture, also taken in 1891 by Frank La Roche, illustrates the horizontal movement of flu gas emissions, primarily CO_2_, from the persistent easterly winds pushing emissions close to the Earth’s surface. Both pictures are publicly available and part of the Frank La Roche Photographs - UW Digital Collections. https://content.lib.washington.edu/larocheweb/index.html. [Accessed December 3, 2024].

For instance, **Figure 1** visually illustrates the CO_2_ emissions spewing into the open air from one of hundreds of 1891 Puget Sound sawmills (La Roche, 1891). Throughout 1891, Puget Sound sawmills burned copious amounts of CO_2_ emitting fuel, milling well over a billion board feet of timber (Vassault, 1892). Further adding to their CO_2_ emissions, the sawmills burned refuse lumber and mill waste on-site due to the land constraints within the bordering Sound (Vassault, 1892). Although no definitive open-air CO_2_ data records exist to document the concentrations in 1891’s Puget Sound atmosphere, it is reasonable to assume that it was significantly elevated above the global ambient level (Idso et al., 2001). Furthermore, the climate and local topography of the Puget Sound region can act as a detainment for CO_2_ emissions (Wang and Ostoja-Starzewski, 2004). Rapid elevation changes from sea level to mountainous terrain, influenced by the nearby Cascade Range, combined with frequent inversions, facilitate the formation of katabatic winds, which elevate CO_2_ gas concentrations (Wang and Ostoja-Starzewski, 2004). Moreover, persistent easterly winds push CO_2_ emissions close to the Earth’s surface and adjacent to vegetation (**Figure 1** inset). Consequently, the Kent agricultural acre-tract site was positioned advantageously, situated directly adjacent to CO_2_ emissions from steam-powered sawmills along the Sound’s shores, making it a beneficiary of CO_2_ FACE within Puget Sound. Finally, considering that hop leaves and strobili can substantially increase carbon yield in response to elevated CO_2_ levels (Bauerle, 2021; 2023), the enhanced atmospheric CO_2_ conditions likely contributed to the increased yield observed that year (Bauerle, 2024).

## 4 Gibberellic acid

Perhaps one of the most influential factors related to the enormous hop yield of 1891 may be the result of gibberellic acid application (Bauerle, 2022). Gibberellic acid, undiscovered at the time, is known to expedite the flowering rate and increase the division and quantity of flower cells in short day plants like hops (e.g. Mutasa-Göttgens and Hedden, 2009). Application of low concentrations of gibberellic acid to hop apical meristems have been shown to result in a twofold increase in the number of hops per plant (Bauerle, 2022). From 1886-1895, a massive influx of Asian immigrants arrived in the Puget Sound region. Their unemployment and population peaked from 1890-1900 due to the completion of the transcontinental railroad (Dearinger, 2017). At the time, the city of Seattle’s labor firm - Quong, Chong & Company- contracted floating populations of Chinese labor to work for hop farmers in 1890’s Puget Sound (Dearinger, 2017). Moreover, they were the predominant seasonal agriculture work force for the Puget Sound “hop craze” of the 1880’s and 1890’s (Kopp, 2016). What is the significance of this labor resource relative to gibberellic acids effects on hop yield*?* Rice, a staple food in Asian cuisine, harbors the gibberellic acid producing fungus *Gibberella fujikuroi*, a fungus that infected greater than 50% of the global rice supply in 1891 (Hori, 1898). In 1926, a Japanese researcher was the first to discover gibberellic acid in rice (Kurosawa, 1926). Subsequently, in 1935, gibberellic acid isolation revealed that *Gibberella fujikuroi* naturally produced gibberellic acid (Yabuta, 1935; Yabuta and Sumiki, 1938). Thus, a hormone that promotes hop flowering, carried by a fungus that infects rice, was unknowingly present in hop yards. Furthermore, since 1865, it has been customary for Asian workers to consume rice balls (onigiri) by hand, inadvertently exposing their skin to *Gibberella fujikuroi* spores (Whitelaw, 2006). Unknowingly, workers could then transmit gibberellic acid via direct contact with the meristematic section of the hop bine when performing the necessary horticulture practice of hand-training each bine to a vertical trellis twine. Thus, training hops to encircle a twine via hands laced with *Gibberella fujikuroi* served as an inadvertent application of gibberellic acid, a now known hop flower promoting hormone.

### 4.1 Gibberellic acids mode of entry

Gibberellic acid effectively enters plant tissue in aqueous solution (McComb, 1964). Therefore, early summer rain showers, a common occurrence in Puget Sound’s summer weather, could have played a key role in facilitating gibberellic acid’s entry into hop tissue. Repeated foliage wetting would further increase the timeframe that gibberellic acid persisted in an aqueous state, allowing it to enter hop meristematic tissue effectively and repeatedly. Alternatively, lanolin, a common skin moisturizer, functions as an additional gibberellic acid tissue infiltration agent (Brian et al., 1954). In the late 1800s, lanolin was commonly used as a moisturizer for chapped agricultural worker hands (e.g. Bag Balm, 1899). It would have been commonplace for hop laborers to apply lanolin to their hands when working with hops due to hop dermatitis, a skin rash caused by manually handling hop bines. Moreover, hop dermatitis is exacerbated when hops are handled under damp weather conditions (Struthers, 1953). Therefore, precipitation events and lanolin may have assisted in gibberellic acid tissue entry.

### 4.2 Exogenous gibberellic acid application

Recently, Bauerle (2022) demonstrated that hop bines have a specific tissue growth stage and application location that optimizes gibberellic acid’s tissue entry and effect on flower production. Although gibberellic acid can enter different types of hop tissue, young meristematic tissue permits maximum absorption into a hop’s vasculature. Furthermore, flower proliferation effects are the most pronounced after exogenous application at low concentrations during the hop juvenile to adult phase transition (Bauerle, 2022). By coincidence, the common horticulture practice of hand-training each hop bine to encircle the trellis twine takes place during the juvenile to adult phase transition. As a result, exogenous gibberellic acid application, together with plentiful plant growth resources, can double the amount of hop strobili per plant (Bauerle, 2022).

## 5 Edge effect and spacing factors

Two additional factors may have added to the hop yield of 1891, hill spacing and edge effects. Hill spacing was approximately 2.13 m, which provided each plant per two plant hill an approximate 2.32 m^2^ of growing area; a generous amount of growing space for commercial hop production as compared to a three or four bine hill (Rybaček, 1991). Next, the size of the hop plot was small, approximately 0.4 hectares. The smaller the plot, the greater the proportion of edge effects relative to the total plant population. Edge effect plants benefit from less plant-to-plant resource competition (e.g. solar radiation penetrating deeper in the canopy). Assuming a 6-meter edge effect from the boundary of a quadrilateral plot, approximately 34% of the total 0.4-hectare cultivation area is affected. Supposing a small plot base yield of 3,800 kg/ha (Haunold et al., 1983), plus 40% for GA_3_ flower proliferation and improved yield under atmospheric CO_2_ enrichment (Bauerle, 2024), 5,320 kg/ha results. After adjusting for the edge effect, 66% of the plot yields 5,320 kg/ha, while the remaining 34% benefits from a 43% yield increase attributable to optimal lighting within the crown, as documented in Bauerle (2024). Thus, the total yield is calculated as (0.66 x 5,320) + (0.34 x 7,607) = (3,511 + 2,586) = 6,097 kg/ha. The yield estimate is near the 1891 record.

## 6 Discussion

Environmental conditions of the Puget Sound ecozone, coupled with a deeper understanding of factors that optimize hop yield (Bauerle, 2021; 2024; Bauerle and Hazlett, 2023), and logical assumptions about events that occurred in 1891 at the Kent hop plot may explain how the hop yield record was achieved. Bauerle (2024) combined supplemental CO_2_ and gibberellic acid application under cool temperatures and high light conditions in a controlled environment. The intention was to simulate the totality of potential hypothesized occurrences that existed at the 1891 Kent hop plot together with the environmental conditions of the Puget Sound ecozone. Although yield did not surpass the record, it came remarkably close: 6,097 dry hop kg/ha versus the record of 6,268 kg/ha (Bauerle, 2024). The recent research discoveries support the notion that the serendipitous combination of optimal climate and growing conditions, elevated carbon dioxide, bio stimulant application, and 1891 events coalesced, producing the hop yield record that persists to this day.
